# A new decision tree for diagnosis of osteoarthritis in primary care: international consensus of experts

**DOI:** 10.1007/s40520-018-1077-8

**Published:** 2018-12-11

**Authors:** Johanne Martel-Pelletier, Emmanuel Maheu, Jean-Pierre Pelletier, Ludmila Alekseeva, Ouafa Mkinsi, Jaime Branco, Pierre Monod, Frédéric Planta, Jean-Yves Reginster, François Rannou

**Affiliations:** 1 Osteoarthritis Research Unit, University of Montreal Hospital Research Centre (CRCHUM), Montreal, Canada; 2 Rheumatology Department, Saint-Antoine Hospital, Assistance Publique–Hôpitaux de Paris (AP-HP), Paris, France; 3 V.A. Nasonova Research Institute Rheumatology, Moscow, Russia; 4 Ibn Rochd University Hospital, Casablanca, Morocco; 5 Department of Rheumatology, NOVA Medical School, CEDOC, Universidade Nova de Lisboa, CHLO, Hospital Egas Moniz, Lisbon, Portugal; 6 Cabinet de Rhumatologie, Castelnaudary, France; 7 Pierre Fabre Consumer Health Care, Castres, France; 8 Department of Public Health, Epidemiology, and Health Economics, University of Liège, Liège, Belgium; 9 Belgium and WHO Collaborating Centre for Public Health Aspects of Musculoskeletal Health and Aging, Liège, Belgium; 10 Service de rééducation et réadaptation de l’appareil locomoteur et des pathologies du rachis, Laboratoire de Pharmacologie, Toxicologie et Signalisation Cellulaire, Univ. Paris Descartes, PRES Sorbonne Paris Cité, Hôpital Cochin, Assistance Publique -Hôpitaux de Paris, INSERM UMR-S 1124, UFR Biomédicale des Saints Pères, Paris, France

**Keywords:** OA, Primary care, Hip, Knee, Hand, Consensus, Care pathways, Diagnosis, General practitioners, Specialist intervention thresholds

## Abstract

**Background and aims:**

Although osteoarthritis (OA) is managed mainly in primary care, general practitioners (GPs) are not always trained in its diagnosis, which leads to diagnostic delays, unnecessary resource utilization, and suboptimal patient outcomes.

**Methods:**

To address this situation, an International Rheumatologic Board (IRB) of 8 experts from 3 continents developed guidelines for the diagnosis of OA in primary care. The focus was three major topologies: hip, knee, and hand/finger OA. The IRB used American College of Rheumatology diagnostic criteria.

**Results:**

Care pathways based on clinical and radiological findings were developed to identify intervention thresholds for GPs/specialists. To optimize usefulness in the primary care setting, the guidelines were formatted as an uncomplicated, but comprehensive one-page decision tree for each topology, highlighting key aspects of the evaluation process and incorporating red flags. In a two-phase validation stage, the draft guidelines were evaluated by rheumatologists and GPs for project execution, content and perceived benefit. The strength of the guidelines lies in their user-friendly diagram and potential for broad application. Such guidelines will allow GPs to make an easy but definite diagnosis of OA and offer clear guidance about situations requiring an expert opinion. The guidelines have potential to improve patient outcomes and reduce the number of unnecessary procedures.

**Discussion and conclusions:**

This project demonstrated the feasibility of developing easy-to-use and effective visual decision trees to facilitate the diagnosis and management of OA of the hip, knee and hand/finger in primary care. The next step should be to conduct a large impact study of implementation of these recommendations in the diagnostic management of OA in general practice in different areas.

## Introduction

Osteoarthritis (OA) is the most common form of arthritis and a leading cause of pain and disability worldwide [1–3]. The most frequently affected peripheral joints are the hip, knee and hand/finger [4]. Risk factors for OA include sex, previous joint injury, obesity and metabolic syndrome, genetic predisposition, mechanical factors such as malalignment or abnormal joint shape, and advancing age [5, 6]. Long regarded as a “degenerative wear and tear” condition, OA is increasingly being recognised as a dynamic joint pathological process caused by destruction and repair for which treatment interventions can be applied.

In 2012, the World Health Organization (WHO) reported that OA is the single most common cause of disability in older adults [7]. Worldwide, an estimated 10% of men and 18% of women over 60 years of age have symptomatic OA; approximately 80% of these have movement limitations and 25% are unable to perform major activities of daily living. With the global increase in the older population, the prevalence of diseases such as OA will also increase. Indeed, by the year 2050, the WHO estimates that 130 million people will have OA and 40 million will be severely disabled by OA [7].

OA is a frequent cause of healthcare consultations. In France, for example, on an annual basis, OA is responsible for approximately 9 million consultations, 14 million prescriptions and 300,000 radiological examinations [http://www.stop-arthrose.org]. In 2010, the total direct costs for treating all patients with OA in France was estimated at about €3 billion per year [8], which emphasizes the burden of the disease to healthcare systems and to society in general. The burden of OA includes not only physical impairment [9] and its associated costs but also psychological impairment (e.g., distress, devalued self-worth) [4]. OA plays a prominent role in multimorbidity, which has been shown to reduce quality of life [10] and to increase work disability, treatment burden and healthcare costs [11]. The disease is also associated with a higher risk of mortality, estimated to be increased by 1.5 in hip and knee OA [12, 13].

Despite the availability of evidence-based treatment guidelines for OA [14], large gaps remain in the overall quality of care. According to patients, pain is generally insufficiently considered and managed [15, 16]. Diagnostic procedures are often inconsistent, and behavioural and rehabilitative strategies to prevent and treat OA are generally underutilized [3]. Uptake of core non-pharmacological measures such as weight loss and exercise programmes tends to be low, especially in older patients (> 65 years of age) even if these treatment modalities have no severe side effects [17].

Besides experiencing pain and loss of function, patients may be frustrated because their disease is not being taken seriously [3, 18, 19]. A Cochrane systematic review suggested that interventions such as improving general practitioner (GP) training regarding OA pain and use of influential physicians may increase guideline-consistent behaviour and improve patient outcomes [20].

Therefore, we need to establish guidelines for the diagnosis of OA in the primary care setting, taking into account barriers to implementing the guidelines as well as possible solutions to overcome these barriers [21–23]. Surprisingly, the OA scientific community had developed several guidelines for OA treatment before establishing clear recommendations for OA diagnosis. This paradox must be changed.

## Methods

### Expert panel

Eight experts from Russia (L.A.), Morocco (O.M.), Canada (J.M-P, J-P.P.), Portugal (J.B.), France (E.M., F.R.), and Belgium (J-Y.R.), selected because of their practical and/or academic expertise in the field of OA, were invited to participate in an International Rheumatologic Board (IRB) set up by Laboratoires Pierre Fabre (Castres, France) with the aim of improving the management of OA in primary care. The expert panel consisted of rheumatologists (L.A., O.M., J.B., E.M., J-P.P), a physical and rehabilitation medicine specialist (J.Y.R), a rheumatologist and physical and rehabilitation medicine specialist (F.R.), and a clinical scientist (J.M-P). All board members were experienced in academic medicine and/or private practice and had expertise in clinical research methodology.

### Guideline development process

The guidelines development initiative was a multistage process. In the first stage, the IRB identified and agreed on OA phenotypes and diagnostic criteria and developed preliminary care pathways for managing OA within the context of a primary care consultation.

The draft guidelines subsequently underwent a two-stage validation process. The first stage involved evaluation of the project by 41 rheumatologists. Rheumatologists from across France who worked in hospitals and/or private practice and with known expertise in managing OA were invited to participate in a reading committee. Questionnaires were used to capture rheumatologists’ opinions on all aspects of the project, including the project execution process, guideline content, and perceived benefit and value of the guidelines. After incorporating rheumatologists’ feedback, a second validation phase was undertaken with 20 GPs in tandem with one of the author. Two meetings were held, one in Toulouse and one in Versailles (both in France). Study Coordinator Dr Pierre Monod and IRB member Professor François Rannou were present at both meetings.

A 54-item questionnaire has been developed during two physical meetings of the IRB in Paris with the help of CLINACT, a French society independent from Pierre Fabre, and expert in clinical research.

A 54-item questionnaire was used to capture the GPs’ level of agreement on the overall usefulness of the project and the content, ease of use and benefits of the guidelines and their practicality for use in daily practice. GPs were invited to propose amendments to the guidelines and provide any additional comments.

## Results

### Establishing the need for guidelines

The need to establish guidelines for the diagnosis of OA in primary care arose from recognising that diagnostic uncertainties exist in this setting and that GPs have limited time to devote to a chronic disease with few effective management solutions. In everyday practice, GPs may be unsure about when to refer patients, often for fear of “bothering” specialists with a “simple” case of OA. Some patients are referred for unnecessary examinations, others at a late stage, and others who could receive primary care. Management delays represent a missed opportunity for the patient and can result in suboptimal outcomes. Moreover, many patients with OA are dissatisfied with their disease burden and with the attitude of “inevitability and fatalism” often held by care providers. The IRB deemed that such guidelines would allow GPs to make an easy but definite diagnosis of OA and offer clear guidance about situations requiring expert opinion. This objective would have potential to improve patient outcomes and reduce the number of unnecessary referrals or procedures.

The guideline development process was validated by the rheumatologist reading committee based on unanimous agreement that early diagnosis of patients with suspected OA needs to be improved (100%) and that improved management would reduce the prescription of unnecessary supplementary examinations such as knee MRI and vascular Doppler (100%). Despite general good agreement by participating rheumatologists that not all patients with suspected OA need to be seen by a specialist, there was some reluctance by rheumatologists to be excluded from the care of these patients, particularly during treatment initiation/optimisation (14.8%). The GPs agreed unanimously that OA assessment must be improved in primary care (100%) and that the board’s procedure was beneficial (100%) and useful (100%).

#### Establishing phenotypes

The main reasons for performing disease phenotyping are to identify specific treatment solutions and monitor the progression of the phenotype over time to adapt management. To this end, the IRB identified four main OA phenotypes:

- Inflammatory OA
- Mechanical OA: trauma, dysplasia, misalignment, overweight and obesity, hypermobility
- Early-onset OA
- Systemic OA: metabolic, microcrystals, hormonal, micro-inflammation

These phenotypes were applied to the three most common peripheral OA localizations: hip, knee, and hand/finger. There was agreement within the IRB to exclude OA of the spine from the project.

### Diagnostic criteria, care pathways and red flags by OA topography: main amendments recommended by rheumatologists and GPs

#### Diagnostic criteria

The IRB used American College of Rheumatology (ACR) diagnostic criteria [24] to develop simple definitions to characterize patients who present in primary care with pain in the hip, knee or hand/finger. For each affected joint, a diagnosis of OA can be made on the basis of joint pain, age over 50 years, and the presence of joint space narrowing and/or osteophyte(s) on plain radiographs, which is the current standard imaging modality in clinical practice for diagnosing and monitoring OA.

Other diagnostic criteria suggested by the rheumatologist reading committee were mainly a physical examination (12.2%) and radiographic assessment (12.2%). The committee also highlighted the need to further elaborate the characteristics of pain. Although most rheumatologists (78.1%) were of the opinion that specialist assessment was not necessary once a diagnosis was made, notable exceptions were for differential diagnosis (22.0%) and cases of ineffective treatment or need for local treatment (14.9%).

After reviewing rheumatologists’ feedback, the IRB took a considered decision not to amend the diagnostic criteria so as to avoid delaying diagnosis and complicating initial management. The definition “abnormal pain of an intensity and duration that would be considered unusual for this disease” was refined by describing intensity (visual analogue scale [VAS] score > 7] and duration (longer than 10 days). The qualifier “magnetic resonance imaging (MRI) is not indicated” was added to the diagnostic criteria for knee pain.

The GPs unanimously agreed that the diagnostic criteria for hip, knee and hand/finger pain were satisfactory (100%) and relevant to primary practice (100%). The GPs also requested greater clarification of the terms “rapidly destructive coxarthrosis (RDC)” and labral anomalies, possibly in an Appendix to the guidelines.

#### Diagnostic algorithm of hip OA in primary care

**Fig. 1:**
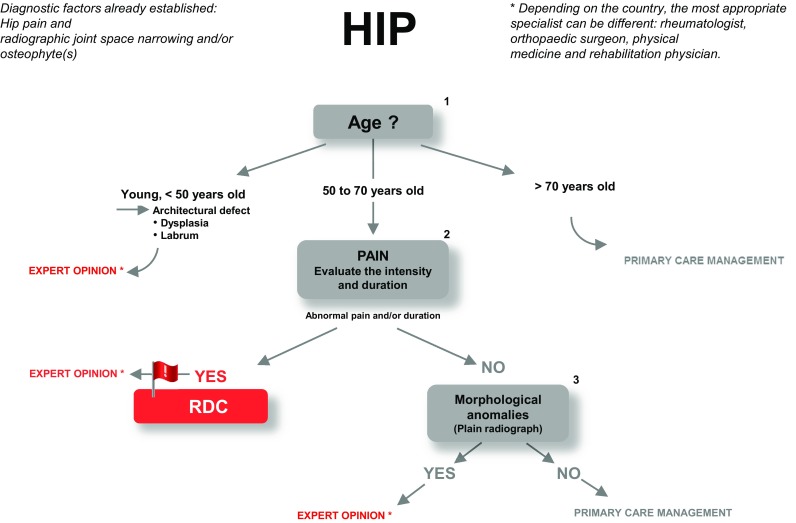
Guidelines for the diagnostic management of hip pain in primary care. Explanatory terminology and acronyms: Abnormal pain: pain of an intensity or duration that is unusual for OA of the hip ; VAS score > 7 or duration longer than 10 days. RDC: rapidly destructive coxarthrosis or microfracture of the subchondral bone responsible for nocturnal pain and limping for which the only effective treatment is unloading body weight from the limb. Labral abnormalities: joint pain sometimes occurring with no radiographic or ultrasound abnormality, most common in young and/or athletic patients, requiring a specialist opinion. Situations requiring urgent expert opinion are denoted by red typeface

Guidelines for OA of the hip are presented in Fig. 1.

The preliminary diagnostic criteria for OA of the hip are hip pain and radiographic joint space narrowing and/or osteophytes.

Additional investigations

- Age

In patients ≤ 50 years of age, investigate an architectural defect (dysplasia, labrum). If present, seek expert opinion.

In patients > 70 years of age, primary care management.

In patients 50 to 70 years of age, investigate the remaining diagnostic criteria in a stepwise fashion.

- Pain

For joint pain of abnormal intensity and/or duration, consider the presence of RDC or a subchondral bone microfracture →red flag. If present, seek expert opinion urgently.

If joint pain is not of abnormal intensity and/or duration, proceed to the next criterion.

- Morphological anomalies

In the absence of morphological anomalies on plain radiographs, manage the OA in primary care. If morphological anomalies are present on plain radiographs, refer the patient for expert opinion.

#### Diagnostic algorithm of knee OA in primary care

**Fig. 2:**
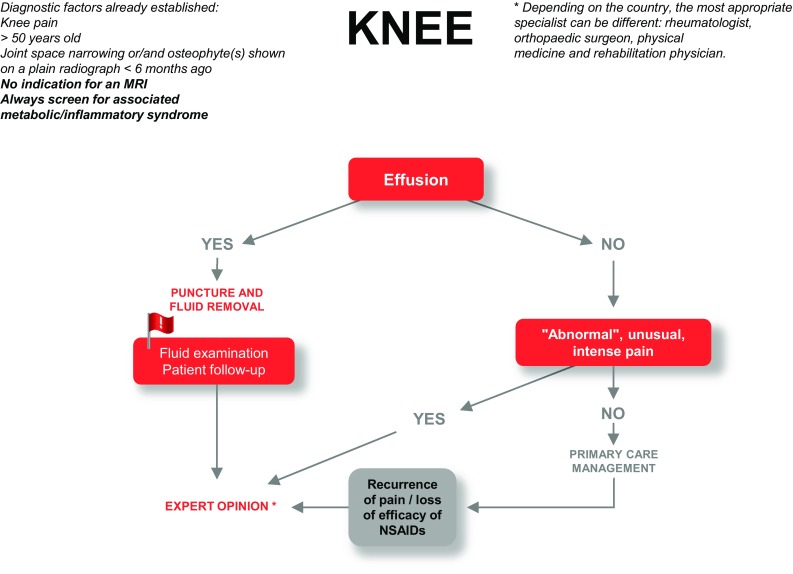
Guidelines for the diagnostic management of knee pain in primary care. Knee radiographs proposed in accordance with the French classification of procedures: anteroposterior weight-bearing, 0° and 30° flexion, profile and 30° flexion skyview (femoro-patellar view). Abnormal pain: pain of an intensity or duration that is unusual for OA of the knee; VAS score > 7 or duration longer than 10 days. Metabolic/inflammatory syndrome assessment: fasting glucose, investigation of an abnormal lipid profile, BMI/overweight, arterial hypertension. NSAIDs: non-steroidal anti-inflammatory drugs. Situations requiring urgent expert opinion are denoted by red typeface

Guidelines for OA of the knee are presented in Fig. 2.

Preliminary diagnostic criteria for OA of the knee are a patient presenting knee pain, > 50 years of age, and radiographic joint space narrowing and/or osteophyte(s) shown on a radiograph performed within the previous 6 months [25] and MRI is not indicated for the first-line diagnosis of knee OA. Associated metabolic/inflammatory syndrome must always be screened.

Additional investigations

- Effusion

The presence of effusion is a red flag. Puncture and drainage of the knee is required, with fluid analysis. The patient is to be referred for expert opinion.

In the absence of effusion, proceed to the next criterion.

- Pain

If “abnormal”, unusual, or intense pain is present on examination, refer the patient for expert opinion. If pain is absent, manage the condition in primary care.

In the event of recurrent pain or loss of efficacy of NSAIDs (i.e., disease progression), refer the patient for expert opinion.

#### Diagnostic algorithm of hand/finger OA in primary care

**Fig. 3:**
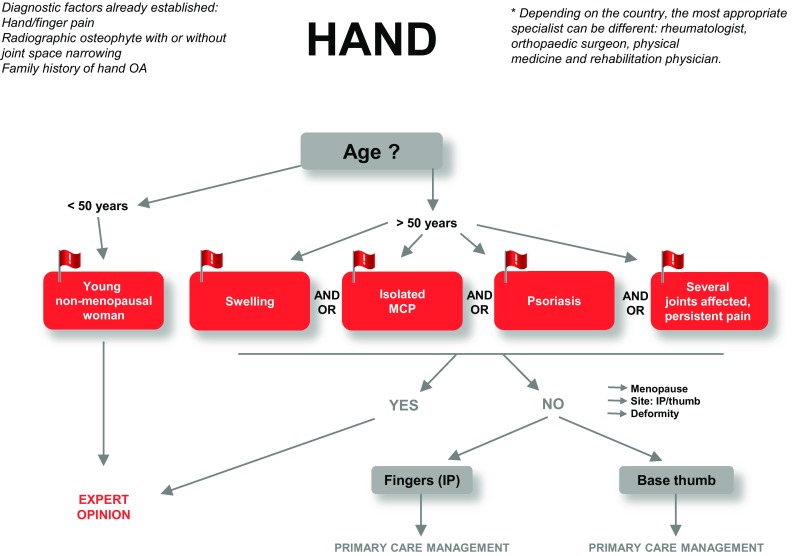
Guidelines for the diagnostic management of hand/finger pain in primary care. IP: interphalangeal; MCP: metacarpophalangeal. Situations requiring urgent expert opinion are denoted by red typeface

Guidelines for OA of the hand/finger are presented in Fig. 3.

Preliminary diagnostic criteria for hand/finger OA are hand/finger pain, radiographic osteophyte with or without joint space narrowing, and a family history of hand/finger OA.

#### Additional investigations

If the patient is female, < 50 years of age, and non-menopausal, refer for an expert opinion. For all other clinical situations, proceed to the next criterion.

The presence of swelling, isolated metacarpophalangeal joint pain, psoriasis, or several joints affected or persistent pain are red flags. If present, refer the patient for expert opinion. If absent, examine the patient clinically for the site of pain and presence of deformity.

Interphalangeal OA must be distinguished from base-of-the-thumb OA because of their different physiopathogenesis.

Although the severity of hand/finger OA is defined by the number of affected joints, no consensus was reached on cut-off points (i.e., number of joints required) to establish grades of severity.

### Key aspects of the validation process informing the development of the guidelines

Globally, the rheumatologist reading committee considered the proposed recommendations for diagnostic management of hip, knee and hand OA in primary care to be logical, easy to understand, and with well positioned GP/specialist thresholds guided by useful red flags.

The main amendments proposed for managing hip pain were to include additional red flags (functional impact, inflammatory pain, inflammatory syndrome) to be more exhaustive. The rheumatologists confirmed that MRI is not indicated for the diagnosis of knee OA and should not be prescribed.

Many rheumatologists (78.1%) agreed with the concept of a more rapid referral of patients presenting hand/finger joint pain. Nearly three-quarters (73.2%) of the group agreed with the IRB’s recommendation that a polyarticular condition warrants an immediate signal alert and proposed to set a severity threshold at 2 or 3 or more affected joints. However, no consensus was reached on this issue.

There was consensus or strong agreement among GPs on key aspects of the care pathways for OA of the hip, knee and hand/finger. The GPs declared that selected entry points met expectations, that specialist intervention thresholds were well positioned with clinically relevant red flags, and that the guidelines would improve the management of OA of the hip, knee and hand/finger in primary practice.

Main comments put forward by GPs about the guidelines for diagnostic management of hip pain centred on identifying strategies to improve their knowledge of RDC and recognising that not all GPs can identify an architectural defect.

In terms of the guidelines for diagnostic management of knee OA, the GPs commented that the proposed care pathway is compromised by prolonged waiting times for specialist appointments, that systematic effusion puncture is not always possible in primary care, and that insufficient emphasis is placed on OA of the knee in patients < 50 years of age, which is commonly encountered in primary care. The GPs emphasized the usefulness of guidelines in terms of educating patients that MRI is unnecessary for first-line diagnosis and management of OA of the knee and expressed considerable interest about their role in screening for associated metabolic syndrome.

Although the diagnostic guidelines for hand/finger pain were considered simpler and more useful than those for hip and knee pain because of entry into care pathways via red flags, the GPs were uncertain as to the justification for some of the red flags. A proposal advanced was to include screening for haematochromatosis for isolated metacarpophalangeal joint disease.

## Discussion

Many clinical practice guidelines are available for managing OA in primary care. Some examples include guidelines from the European Society for Clinical and Economic Aspects of Osteoporosis and OA [14], the American Academy of Orthopedic Surgeons [26], ACR [27], Chinese Orthopedic Association [28], European League Against Rheumatism [29, 30], National Institute for Health and Clinical Excellence [31] and OA Research Society International [32]. Despite the uniformly high quality of these guidelines, their implementation in clinical practice has been low [22]. Barriers to guideline implementation are complex and multifactorial, ranging from evidence limitations and human behaviour to presentation in an inappropriate format to the end user [21, 22]. As a rule, primary care physicians prefer shorter formats, such as flowcharts or algorithms and single-page checklists, over longer formats [23].

The strengths of the IRB’s guidelines for diagnosis of OA in primary care lie in their user-friendly diagrams. A diagnosis of OA can be confirmed by the presence of three simple ACR criteria without the need to seek specialist opinion or perform further investigations. The care pathways map a logical approach to managing the diagnosed patient, incorporating only strictly necessary physical examinations, clinical criteria, and specialist intervention thresholds into the management algorithm. The convenient and informative one-page graphical format is designed to facilitate normal work flow in primary care and lends itself to future integration into prescribing software. Moreover, the guidelines have been developed by an international panel of experts for application across a wide range of countries and healthcare systems.

The purpose of developing guidelines for the diagnosis of OA in primary care was to provide GPs with an easy and effective tool to facilitate the diagnosis of OA and to improve care of the diagnosed patient. To ensure the exhaustiveness of the clinical situations addressed in our guidelines, validate their content and confirm their utility in the primary care setting, the project execution process was iterative, informed in two separate stages with feedback after evaluation by rheumatologists and primary care physicians.

Although the reading committee approved the justification for developing guidelines to improve the diagnosis of OA in primary care (i.e., to improve patient care) and acknowledged the importance of GP involvement, some concerns were raised about GPs’ level of knowledge in certain areas (e.g., their ability to differentiate joint pain from tendinitis and to interpret radiographs) and about removing the rheumatologist from the centre of patient care, especially for situations such as the differential diagnosis and progressive disease. There were some reservations about excluding OA of the spine and some support for developing guidelines for OA of the ankle, foot and shoulder. The simple bases for diagnosing OA also drew some criticism. In particular, a frequent comment was that pain should be investigated in more detail in terms of type, site and nature (referred, mechanical or inflammatory).

The guidelines were well received by GPs who were in strong agreement about their ease of use, potential benefit and practicality for diagnosing and managing OA of the hip, knee, or hand/finger in primary care. The three selected OA sites were considered relevant, although the shoulder was mentioned as another site to consider. A consistent finding during the GP validation stage was that for each of the OA sites, less than half of the group believed that the guidelines reflected current clinical practice, whereas most of the group believed that these guidelines would improve the diagnostic management of OA. The inference is that current practice may be suboptimal and that our guidelines have the potential to improve current practice, which is a core objective of any guideline development initiative. During the course of evaluation, the GPs identified certain areas in which they may be lacking expertise (e.g., diagnosis of RDC, identification of an architectural defect). Such insight is valuable in terms of informing continuing education programmes and other activities aimed at upskilling primary care physicians.

The feedback received after consultation with rheumatologists and GPs validated most aspects of these guidelines and informed several important amendments. The relatively minor changes to the diagnostic criteria from the first draft to the final version confirmed the decision to use the validated ACR criteria. Specifying the intensity and duration of hip and knee pain that would be considered unusual for the setting was a small but important change that eliminated ambiguity and has the potential to limit referrals to the neediest cases. Transferring the responsibility for caring for patients > 70 years of age without abnormal pain and morphological anomalies from specialist care to primary care reflects not only the global trend in population ageing (and associated increase in the prevalence of OA) but also the general better health and well-being of older individuals as compared with previous generations. Significant cost-savings might be expected to accrue from caring for otherwise healthy older adults with OA of the hip in primary care. An important element of the care pathway for OA of the knee is the indication to systematically screen patients for the presence of metabolic/inflammatory syndrome (fasting glucose, lipid profile, overweight, hypertension). Such screening is common and appropriate in primary care and can identify modifiable risk factors for a wide range of diseases. The addition of “MRI is not indicated” in the diagnostic criteria for suspected OA of the knee was highly valued by GPs as a means of educating patients that MRI is not necessary for a first-line diagnosis. This subtle yet strong message has potential to generate significant time and cost-savings and is consistent with objectives to expedite diagnoses and eliminate unnecessary examinations [33].

The guideline development project has several limitations. Among 665 rheumatologists invited to participate in the reading review, only 41 (6.2%) returned the questionnaire. The main reasons for not participating were lack of time, the voluntary aspect of survey, and a general lack of interest in studies or surveys. Thus, although the reading committee was in reasonably strong agreement about the benefit of the proposed recommendations, this low response incorporates a degree of bias because it reflects the opinion of only those rheumatologists with an interest in the subject and who agreed to participate. It might be assumed that their participation was linked to this particular interest in the subject and to the issues covered by the recommendations. Another limitation was the modest number of GPs who participated in the validation phase (*n* = 13), so the representativeness of the sample cannot be guaranteed. A larger validation study would help ensure the validity of our guidelines and might be useful to promote their wide use. Although OA can occur in any joint, the guidelines cover only the three major peripheral topographies. This was recognised as a limitation by the rheumatologists and GPs who suggested other sites (e.g., spine, shoulder) that could benefit from expert guidance. Finally, we acknowledge that the guidelines provide limited recommendations for treatment approaches, pharmacological, non-pharmacological and other (e.g., lifestyle advice); however, (1) this was not the primary aim of our work and (2) this compromise was deemed necessary to keep within the one-page format and is compensated to some degree by the user-friendly interface showing essential GP/specialist intervention thresholds.

## Conclusions

This guidelines project has demonstrated the feasibility of developing easy-to-use and effective visual decision trees to facilitate the diagnosis of OA of the hip, knee and hand/finger in primary care. The next step should be to conduct a large impact study of implementation of these recommendations in the diagnostic management of OA in general practice in different areas.

## Appendix : Evaluation of the process

### Evaluation questionnaire

#### Evaluation of the process

**Table:**

Q1	Do you agree with the board’s statement that osteoarthritis is managed inefficiently in primary care?	A: YES B: NO
Q2	If yes, do you agree that the way in which the disease is managed in primary care needs to be improved?	A: YES B: NO
Q3	Do you agree that delayed management of osteoarthritis can represent a missed opportunity for the patient?	A: YES B: NO
Q4	A simple and well-established procedure for GPs to apply would be useful to help them better manage osteoarthritis	A: TRUE B: FALSE
Q5	Offering GPs a simple procedure to apply would reduce the prescription of unnecessary additional examinations?	A: TRUE B: FALSE
Q6	The creation of osteoarthritis guidelines is based on the idea that not all patients with suspected osteoarthritis necessarily need to be seen by a specialist. Do you agree with this?	A: YES B: MOSTLY YES C: MOSTLY NO D: NO
Q7	Do you agree with the fact that osteoarthritis of the spine has been excluded from these primary care guidelines?	A: YES B: NO
Q8	Do you consider it relevant that the guidelines are focused on the three sites of the hip, knee and hands?	A: YES B: NO
Q9	Do one or more of the three guidelines seem unnecessary to you?	A: YES B: NO If yes, which one(s)?
Q10	Do you believe other osteoarthritis sites should be priorities in primary care patient management guidelines?	A: YES B: NO If yes, which one(s)?
Q11	Are there any situations in which excluding the rheumatologist from the disease’s management could have a negative impact on the patient?	A: YES B: NO If yes, which one(s)?

#### Evaluation of the content of the guidelines

- Concerning the proposed basis for diagnosis

**Table:**

Q12	Do you agree with the proposed diagnosis criteria for GPs?	A: Yes B: NO
Q13	If no, which criteria would you have proposed in order for GPs to perform an osteoarthritis diagnosis on one of the three sites?	Open answer
Q14	Once osteoarthritis has been diagnosed on one of the three sites, would you consider it essential for the patient to receive a specialist assessment by a rheumatologist?	A: YES B: NO
Q15	If yes, what should this assessment include?	Open answer
Q16	If no, are there any situations in which a specialist assessment would be necessary? (other than the alert situations described in the guidelines)	Open answer
Q17	For surgical osteoarthritis, who do you believe would be the best placed practitioner to manage the patient?	A: The GP B: The surgeon C: The rheumatologist
Q18	For osteoarthritis requiring medical treatment, who do you believe would be the best placed practitioner to manage the patient?	A: The GP B: The rheumatologist
Q19	In your opinion, which of the following statements is most accurate	
	For newly diagnosed osteoarthritis, a rheumatologist’s opinion is essential	A
	For newly diagnosed osteoarthritis, a rheumatologist’s opinion would be useful, only in a complex situation or if treatment fails	B
	Except in specific situations, osteoarthritis should be treated exclusively by a GP	C

- Concerning the procedure to apply for osteoarthritis of the hip

**Table:**

Q20	On the whole, the procedure is logical	YES NO
Q21	On the whole, the procedure is easy to understand	A: YES B: NO
Q22	The red flags are useful for this indication	A: YES B: NO
Q23	I believe other red flags would be useful	A: YES B: NO If Yes, please specify which ones
Q24	The intervention threshold between the GP and the specialist seems well positioned	A: YES B: NO
Q25	Only offering a plain radiograph to investigate hip pain in primary care seems appropriate	A: YES B: NO
Q26	If you answered NO to question 25, what further investigations would you advise **a GP** to perform?	Open answer
Q27	Would you say then that this proposed procedure for osteoarthritis of the hip is	
	Sufficient for GPs	A
	Goes too far for GPs	B
	Does not go far enough for GPs	C
Q28	Do you have any comments on this procedure for managing osteoarthritis of the hip?	

- Concerning the procedure to apply for osteoarthritis of the knee

**Table:**

Q29	On the whole, the procedure is logical	A: YES B: NO
Q30	On the whole, the procedure is easy to understand	A: YES B: NO
Q31	The red flags are useful for this indication	A: YES B: NO
Q32	I believe other red flags would be useful	A: YES B: NO
Q33	The intervention threshold between the GP and the specialist seems well positioned	A: YES B: NO
Q34	Only offering a plain radiograph to investigate knee pain in primary care seems appropriate	A: YES B: NO
Q35	If you answered NO to question 34, what further investigations would you advise a GP to perform?	Open answer
Q36	Would you say then that this proposed procedure for osteoarthritis of the knee is	
	Sufficient for GPs	A
	Goes too far for GPs	B
	Does not go far enough for GPs	C
Q37	Do you have any comments on this procedure for managing osteoarthritis of the knee?	

- Concerning the procedure to apply for osteoarthritis of the hand

**Table:**

Q38	On the whole, the procedure is logical	A: YES B: NO
Q39	On the whole, the procedure is easy to understand	A: YES B: NO
Q40	The red flags are useful for this indication	A:YES B: NO
Q41	It is logical to position the red flags from the start with osteoarthritis of the hand, owing to the differential diagnoses that could be envisaged	A: YES B:NO
Q42	I believe other red flags would be useful	A: YES B: NO If YES, please specify which ones
Q42a	The fact that several joints are affected constitutes a severity factor	A: TRUE B: FALSE
Q42b	How many hand joints need to be affected for you to consider question 42a to be true?	Quantified answer
Q43	The intervention threshold between the GP and the specialist seems well positioned	A: YES B: NO
Q44	Only offering a plain radiograph to investigate hand/finger pain in primary care seems appropriate	A: YES B: NO
Q45	If you answered NO to question 44, what further investigations would you advise a GP to perform?	Open answer
Q46	Would you say then that this proposed procedure for osteoarthritis of the hand is	
	Sufficient for GPs	A
	Goes too far for GPs	B
	Does not go far enough for GPs	C
Q47	Do you agree that a patient presenting with pain in the hand or finger joints should be referred to a specialist more quickly?	A: YES B: NO
Q48	Do you have any comments on this procedure for managing osteoarthritis of the hand?	

#### Evaluation of the benefit and purpose of the guidelines

**Table:**

Q49	Do you find this tool practical? (you consider this tool to be suited for use in GP consultations)	A: YES B: NO
Q50	Does this tool seem easy to use? (this document is considered easy if it can be used by primary care doctors without complicating their relationship with the patient)	A: YES B: NO
Q51	Do you agree with the board that a lower specialist intervention threshold is needed for osteoarthritis of the hands? (you consider the risk of a differential diagnosis to be high)	A: YES B: NO
Q52	Do you believe that use of these guidelines by GPs could Improve patient management Speed up diagnosis Improve the GP-specialist relationship (for instance, by obtaining an urgent opinion following the application of these guidelines)	YES/NO for each question
Q53	Do you have any further comments that could improve our work?	
Q54	Do you wish to be notified of the progress of this work?	YES/NO
